# Urinary myiasis; a case report and literature review

**DOI:** 10.1016/j.eucr.2025.102992

**Published:** 2025-02-24

**Authors:** Siavash Vaziri, Zeinab Mohseni Afshar, Mohammad-Bagher Rajabalian, Behzad Narouie, Babak Sayad, Seyed Hamid Madani, Zohreh Bartani, Mehdi Sedighi, Negar Radpour, Hamidreza Momeni

**Affiliations:** a Clinical Research Development Center, Imam Reza Hospital, Kermanshah University of Medical Sciences, Kermanshah, Iran; b Department of Urology, Babol University of Medical Sciences, Babol, Iran; c Department of Urology, Zahedan University of Medical Sciences, Zahedan, Iran; d Molecular Pathology Research Center, Emam Reza Hospital, Kermanshah University of Medical Sciences, Kermanshah, Iran; e Department of Urology, Urology and Nephrology Research Center, Shahid Labbafinejad Medical Center, Shahid Beheshti University of Medical Sciences, Tehran, Iran

**Keywords:** Urinary myiasis, fly larvae, Diagnostic challenges, Nonspecific symptoms

## Abstract

Urinary myiasis, a rare condition caused by the infestation of fly larvae in the urinary tract, poses diagnostic challenges due to its nonspecific symptoms and low prevalence. We report a 52-year-old woman with persistent dysuria, frequency despite multiple treatments for suspected infections. Cystoscopy revealed erythema and debris, but imaging and laboratory tests were unremarkable. A live larva was identified in urine analysis, confirming the diagnosis. Treatment involved improved hygiene and hydration. Prompt recognition and management is needed to prevent complications. Awareness of this rare condition is crucial, particularly in individuals with poor sanitation and underlying urinary tract abnormalities.

## 1 Introduction

Human myiasis is defined as infestation of body organs and tissues with the larvae of the dipterous family. The most common species of flies that cause myiasis include Fannia scalaris, Musca, Sarcophaga, Lucilia, Wohlfahrtia and Calliphora. Myiasis may be obligatory, facultative or accidental. The Classification of myiasis is based on the body organ involved. Urinary myiasis is one of the least common types of myiasis as the associated entry sites are usually covered with clothes, making it, inaccessible to insects. It usually occurs in low socioeconomic conditions with poor personal hygiene.^1^^,^^2^ The clinical significance of urinary myiasis is caused by larval growth and toxin secretion, invasion, associated inflammation and progressive necrosis of the bladder wall. Hence, timely diagnosis and management is necessary.^3^ Here, we report a case of urinary myiasis in Iran.

## 2 Case presentation

A 52-year-old woman referred to the urology clinic with urinary complaints. Her symptoms began three years ago with frequency, dysuria and dribbling. She also mentioned the frequent passage of red and black thread-like substances in her urine. Moreover, during these discharges, she had headache, fever and chills. Intermittent periurethral and genital itching was another complaint of hers. She had been treated by several specialists with the diagnosis of recurrent urinary tract infections, with no clinical improvement. The patient denied recent travel, camping, hiking, farming, swimming and insect bites. She had positive history of pilonidal sinus surgery and hysterectomy, 8 and 7 years earlier, respectively. Two years prior to the current visit, she had been hospitalized for assessment. On physical examination, she was well-appearing with normal vital signs. All her laboratory tests, including cell blood count, urine analysis and biochemistries were in normal ranges. Abdominopelvic computed tomography (CT) scan revealed no abnormalities. Hence, she underwent cystoscopy, which demonstrated erythema and hyperemia of the bladder mucosa, suspended debris, and dilation of the left ureteral orifice. During consultation with an infectious diseases’ specialist, schistosomiasis was suspected, Therefore, she was treated with Praziquantel with the appropriate dose and duration and was discharged from the hospital.

However, her symptoms did not subside. She was revisited by another infectious disease specialist, who prescribed her ivermectin due to suspicion to urinary myiasis. Nevertheless, no improvement was observed. She was readmitted to undergo bladder irrigation with polyethylene glycol, but the bladder washfluid did not contain any visible larvae. This procedure was followed by a two-day hematuria with spontaneous cessation. She was discharged home and advised to repeat the urine analysis one months later. Her random urine analysis was normal, so she collected her 24-h urine and sent it to the laboratory for analysis, in which a live larva was demonstrated under light microscope by the pathologist. The larva was isolated and sent to an entomologist to be identified morphologically. Finally, it was determined that the larva belonged to the species Sarcophaga. The patient was advised to take personal hygiene and consume at least 3 L of water daily.

## 3 Discussion

Contamination of the human urinary tract with flies’ larvae is known as urinary myiasis. Urogenital myiasis is a rare type of human myiasis reported in poor sanitary conditions or individuals with underlying urinary tract abnormality or surgical intervention.^4^^,^^5^

The flies are attracted to oviposit around the urethral orifices or external genitalia of humans by urogenital discharges or fecal soiling of the perineal area. Subsequently, the larvae are hatched and pass through the urethra to reach the bladder, leading to urethritis and cystitis.^6^

Clinical manifestations of urinary myiasis include general symptoms (e.g. abdominal pain, nausea, vomiting, itching and rectal bleeding) and specific symptoms (e.g. hematuria, frequency, dysuria, urethral discharge and notification of larvae in the urine); nevertheless, some individuals remain asymptomatic indefinitely.^7^ Tenderness and erythema of the external genitalia may also be reported in female patients.^8^

Due to the nonspecific symptoms, urinary myiasis can be misdiagnosed as other conditions; as such we can mention urethral stone or obstruction, malignancies and schistosomiasis.^9^

Laboratory abnormalities that may be found in urinary myiasis are nonspecific and include proteinuria, hematuria, and leukocyturia,^10^ none of which were present in our patient.

**Fig. 1:**
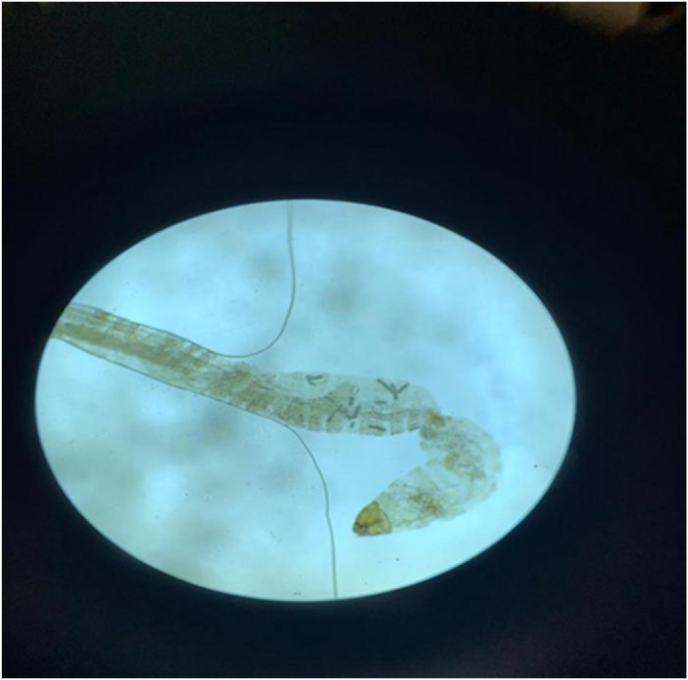
Morphologic examination of the larvae under stereoscopic microscope.The larva belonged to the species Sarcophaga.

Due to the deep-seated location of urinary myiasis, invasive or semi-invasive procedures like cystoscopy and urethroscopy may be needed to extract the larvae. Demonstration of the larvae with head, segments, dorsal and ventral surfaces, spiracles and spines can help in identifying myiasis.^11^ Microscopic examination can reveal larvae bodies consisting of a triangular head with two hairy antennae and multiple thoracic and abdominal hairy and cylindrical segments,^12^ which was true for our patient (Fig. 1). However, the definite diagnosis is based upon molecular tests which determine the larval species.^13^

Treatment choice of urinary myiasis depends on the location of the larvae and the severity of the symptoms. Medical therapy usually fails as no exact pharmacological agent is effective on the larvae. Thus, mechanical removal is the best therapeutic intervention. Nevertheless, antibiotics and anti-inflammatory agents may temporally relieve the symptoms.^14^^,^^15^

## CRediT authorship contribution statement

**Siavash Vaziri:** Conceptualization, Funding acquisition, Methodology. **Zeinab Mohseni Afshar:** Conceptualization, Investigation, Writing – review & editing. **Mohammad-Bagher Rajabalian:** Data curation, Formal analysis, Writing – review & editing. **Behzad Narouie:** Conceptualization, Project administration, Writing – review & editing. **Babak Sayad:** Data curation, Investigation, Methodology, Writing – original draft. **Seyed Hamid Madani:** Conceptualization, Formal analysis, Writing – original draft. **Zohreh Bartani:** Resources, Supervision, Validation, Writing – review & editing. **Mehdi Sedighi:** Conceptualization, Investigation, Methodology. **Negar Radpour:** Conceptualization, Resources, Writing – review & editing. **Hamidreza Momeni:** Data curation, Formal analysis, Methodology.

## Informed consent

Written informed consent was obtained from the patient(s) for their anonymized information to be published in this article.

## Ethics approval

Our institution does not require ethical approval for reporting individual cases or case series.

## Data availability

The data used to support the findings of this study are included within the article.

## Funding statement

N/A.

## Conflict of interest

The authors declare no competing interest regarding the publication of this article.
